# In vivo assessment of the antiparasitic effects of *Allium sativum* L. and *Artemisia absinthium* L. against gastrointestinal parasites in swine from low-input farms

**DOI:** 10.1186/s12917-024-03983-3

**Published:** 2024-04-01

**Authors:** Mihai-Horia Băieş, Vlad-Dan Cotuţiu, Marina Spînu, Attila Mathe, Anamaria Cozma-Petruț, Sorana D. Bolboacǎ, Ricarda Margaret Engberg, Anne Collin, Vasile Cozma

**Affiliations:** 1 Department of Parasitology and Parasitic Disease, Faculty of Veterinary Medicine, University of Agricultural Sciences and Veterinary Medicine of Cluj-Napoca, 3-5 Mǎnǎştur Street, 400372 Cluj-Napoca-Napoca, Romania; 2 Department of Infectious Diseases, Faculty of Veterinary Medicine, University of Agricultural Sciences and Veterinary Medicine of Cluj-Napoca, 3-5 Mǎnǎştur Street, 400372 Cluj-Napoca-Napoca, Romania; 3 Agricultural Research and Development Station of Turda, 27 Agriculturii Street, 401100 Turda, Romania; 4 Department of Bromatology, Hygiene, Nutrition, Faculty of Pharmacy, “Iuliu Haţieganu” University of Medicine and Pharmacy, 6 Pasteur Street, 400349 Cluj-Napoca-Napoca, Romania; 5 Department of Medical Informatics and Biostatistics, “Iuliu Haţieganu” University of Medicine and Pharmacy, 6 Louis Pasteur Street, 400349 Cluj-Napoca-Napoca, Romania; 6 Department of Animal and Veterinary Sciences, Aarhus University, 20 Blichers Allé, 8830 Tjele, Denmark; 7 INRAE, Université de Tours, BOA, 37380 Nouzilly, France; 8 Academy of Agricultural and Forestry Sciences Gheorghe Ionescu-Siseşti (A.S.A.S), 61 Mǎrǎşti Boulevard, 011464 Bucharest, Romania

**Keywords:** Garlic, Wormwood, Low-input farms, Gastrointestinal parasites

## Abstract

**Background:**

Ethno-veterinary practices could be used as a sustainable developmental tool by integrating traditional phytotherapy and husbandry. Phytotherapeutics are available and used worldwide. However, evidence of their antiparasitic efficacy is currently very limited. Parasitic diseases have a considerable effect on pig production, causing economic losses due to high morbidity and mortality. In this respect, especially smallholders and organic producers face severe challenges. Parasites, as disease causing agents, often outcompete other pathogens in such extensive production systems. A total of 720 faecal samples were collected in two farms from three age categories, i.e. weaners, fatteners, and sows. Flotation (Willis and McMaster method), modified Ziehl–Neelsen stained faecal smear, centrifugal sedimentation, modified Blagg technique, and faecal cultures were used to identify parasites and quantify the parasitic load.

**Results:**

The examination confirmed the presence of infections with *Eimeria* spp., *Cryptosporidium* spp., *Balantioides coli (*syn. *Balantidium coli), Ascaris suum*, *Oesophagostomum* spp., *Strongyloides ransomi*, and *Trichuris suis*, distributed based on age category. A dose of 180 mg/kg bw/day of *Allium sativum* L. and 90 mg/kg bw/day of *Artemisia absinthium* L. powders, administered for 10 consecutive days, revealed a strong, taxonomy-based antiprotozoal and anthelmintic activity.

**Conclusions:**

The results highlighted the therapeutic potential of both *A. sativum* and *A. absinthium* against gastrointestinal parasites in pigs. Their therapeutic effectiveness may be attributed to the content in polyphenols, tocopherols, flavonoids, sterols, sesquiterpene lactones, and sulfoxide. Further research is required to establish the minimal effective dose of both plants against digestive parasites in pigs.

**Supplementary Information:**

The online version contains supplementary material available at 10.1186/s12917-024-03983-3.

## Background

In pig farming, management and preventive measures against parasitic diseases improve overall feed conversion and reproductive performance, while decreasing morbidity and mortality [1, 2]. Gastrointestinal parasites pose a significant economic burden on pig farms, leading to various detrimental effects. These include inefficient feed consumption, poor growth rate, reduced weight gain, decreased litter size, fertility disorders, compromised post-vaccination immunity against infectious diseases, lower meat quality, and diminished animal welfare [1, 3, 4].

The diversity and intensity of gastrointestinal parasitism depends on the type of swine production system [3, 5]. Low-input farming faces several constraints, among which parasitic diseases are of significant importance [6]. The prevalence of digestive parasites in pigs is broadly reported upon worldwide. The most common helminths are *Ascaris suum, Trichuris suis*, *Strongyloides ransomi*, *Hyostrongylus rubidus, Trichostrongylus axei*, and *Oesophagostomum spp*. Furthermore, pigs can also harbour intestinal protozoan parasites, such as *Eimeria* spp., *Cystoisospora suis*, *Cryptosporidium spp.*, *Balantioides coli*, and *Giardia lamblia* [1, 7, 8].

Controlling parasite infections in livestock farming is increasingly crucial globally. Antiparasitic medications, like avermectins, triazine, and benzimidazole, are commonly used to combat these parasitic infections in swine [5, 9]. Their primary drawback is the emergence of antiparasitic resistance across most compounds, coupled with the presence of residues in animal products. The residues of such chemicals in the environment can disrupt ecosystems, posing significant threats to human health and welfare [10].

There is renewed international interest in using herbal products as safer alternatives to control parasite infections and lower the risk of developing resistance to antiparasitic drugs [11]. Ethno-medicine holds an integral position within traditional medical practices in numerous developing countries. This type of empirical medicine is mainly used in rural areas, where standard treatment protocols, especially for livestock, have prohibitive costs [12]. Ethno-veterinary practices have the potential to serve as sustainable development tools by leveraging local veterinary and husbandry knowledge. Thus, herbal medicine may very well become a frontrunner in turning local into global knowledge through the recognition of local expertise as an essential source of wide-reaching sustainable development for both people and animals [4, 13]. Over the last decade, there has been a notable increase in the utilization of phytotherapeutic remedies, which can be attributed to their enhanced bioavailability, reduced toxicity, and environmentally friendly characteristics [14].

*Allium sativum* L., commonly known as garlic, belongs to the Amaryllidaceaa family [15, 16]. The plant forms a bulb that is commonly used either as food or medicine. Garlic contains several enzymes, 17 amino acids, along with minerals and more than 33 sulfur compounds, a content higher than in any other *Allium* species. The latter are responsible for both the garlic’s pungent odour as well as many of its medicinal effects [16–18]. The bioactive compounds of garlic are allicin, alliin, ajoene, diallyl sulfide, dithiin, vinyldithiins, and allylcysteine [11, 18, 19]. Dried, powdered garlic contains approximately 1% alliin (S-allyl cysteine sulfoxide). The main anthelmintic compound, allicin (diallyl thiosulfinate or diallyl disulfide) only becomes available in garlic once the bulb is crushed, through activation of the enzyme alliinase, which then metabolizes alliin into allicin [16–18]. Garlic has a wide range of properties, such as antibacterial, antiviral, antifungal, antiprotozoal, and anthelmintic. It can also act as an immune stimulating agent, while reducing the risk of cardiovascular disease [16, 20].

*Artemisia absinthium* L., commonly known as wormwood, is a species belonging to the *Artemisia* genus, distributed mainly in the temperate zone [21]. It contains various compounds, such as: sesquiterpene lactones, essential oils (thujones, trans-sabinyl acetate, cis-chrysanthenyl-acetate, and cis-epoxyimene), along with phenolic acids, flavonoids, carotenoids, coumarins, thiophene, tannins, and lignans [21, 22]. *A. absinthium* and its extracts have several therapeutic properties such as: antioxidant, anticarcinogenic, immuno-modulatory, anti-inflammatory, antipyretic, cardio-protective, gastro-protective, hepato-protective, hypoglycaemic, neuroprotective, and antidepressant [23–25]. In traditional medicine, *A. absinthium* has been used in both humans and animals as an anthelmintic and antiprotozoal drug and is still regarded as an effective natural remedy against parasites [21, 22, 24]. Sesquiterpene lactones, such as artemisinin, dihydroartemisinin, ridentin, hanphyllin, dehydroleucodine, and santonin, constitute the most important molecules responsible for the antiparasitic activity of plants from the *Artemisia* genus [26–28]. In the poultry industry, the bulb of *A. sativum* and whole plant of *A. absinthium* have been used as phytogenic growth promoters, by stimulating the secretion of digestive enzymes, leading to enhanced digestion and absorption [29, 30].

Due to continuously increasing drug resistence in parasites and prohibited use of antiparasitic medications in organic pig farming practices, phytotherapy could represent a valid, biologically available and cost effective alternative for parasite control. Since numerous plants are cited for their antiparasitic effects in different animals species and many of them are commonly locally available, this study focused on exploring the antiparasitic potential of garlic (*Allium sativum*) and wormwood (*Artemisia absinthium*) plants native to Romanian’s flora and renown for their manifold beneficial properties against naturally occurring gastrointestinal parasites of pigs on two low-input (free-range) farms from NW Romania. The primary objective of this research was to identify a plant-based formula that exhibits effectiveness in combating pig parasitoses without interfering with their welfare and health.

## Results

### Chemical analysis of plant extracts

Following chemical analysis of the alcoholic plant extracts, the main biologically active compounds identified were: polyphenols (caffeic acid, ferulic acid, sinapic acid), tocopherols (α-tocopherol), sulfoxide (aliin) for *A. sativum* and polyphenols (chlorogenic acid, *p*-coumaric acid, ferulic acid, vitexin, isoquercitrin, rutoside, quercitrin, quercetol, luteolin, kaempferol, apigenin, syringic acid, protocatechuic acid, and vanillic acid), tocopherols (α-tocopherol, γ-tocopherol, Δ-tocopherol), sterols (ergosterol, stigmasterol, β-sitosterol, campesterol), methoxylated flavones (hispidulin, eupatorin, casticin), sesquiterpene lactones (α-santonin, vulgarin) for *A. absinthium*.

### Evaluation of plants’ antiparasitic activity

In this experiment, the animals readily consumed the feed without any hesitation and did not exhibit any noticeable adverse effects. Although it was not the primary objective of the study, clinical observations indicated that animals in both experimental groups across all age categories showed enhanced feed intake and a higher growth rate compared to those in the control groups. This effect was particularly evident among the weaners and fatteners.

The coproparasitological examination revealed co-infections with seven species of gastrointestinal parasites, including *Eimeria* spp., *Balantioides* coli, *Cryptosporidium* spp, *Ascaris suum*, *Trichuris suis*, *Oesophagostomum* spp., and *Strongyloides ransomi*, in combinations depending on the category of pigs. Oocysts culture examination successfully attributed the parasites to the *Eimeria* genus. Faecal cultures, containing strongylid eggs, showed that all L3 larvae belonged to the *Oesophagostomum* genus. Neither the centrifugal sedimentation nor Blagg methods revealed positive results. The flotation, oocysts/egg culture, and McMaster methods showed that the prevalence and the average intensity of infections varied according to farm, age category, and tested plant.

**Fig. 1:**
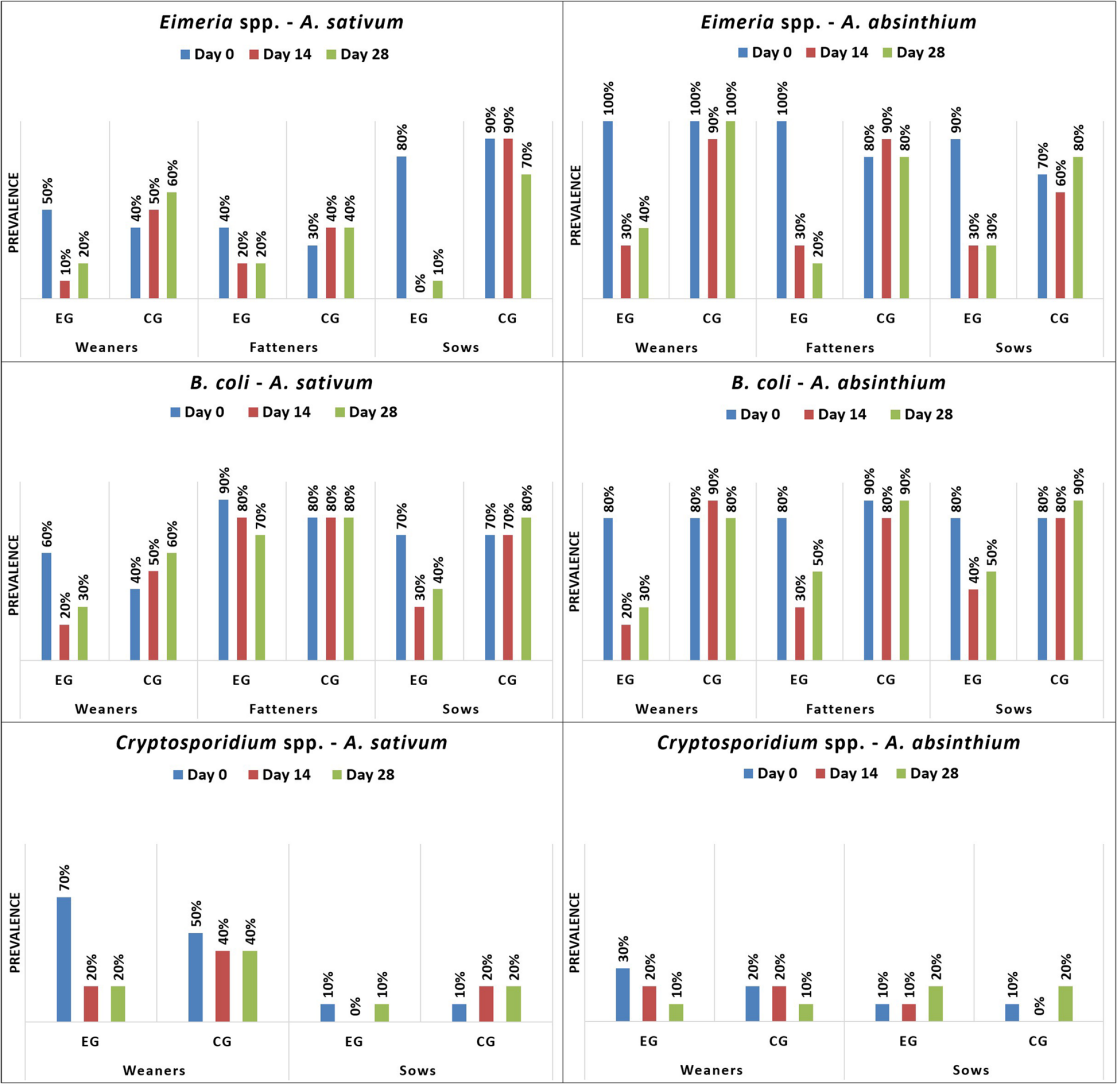
Prevalence (%) of investigated protozoa on farm 1 by age group (EG = experimental group; CG = control group)

**Fig. 2:**
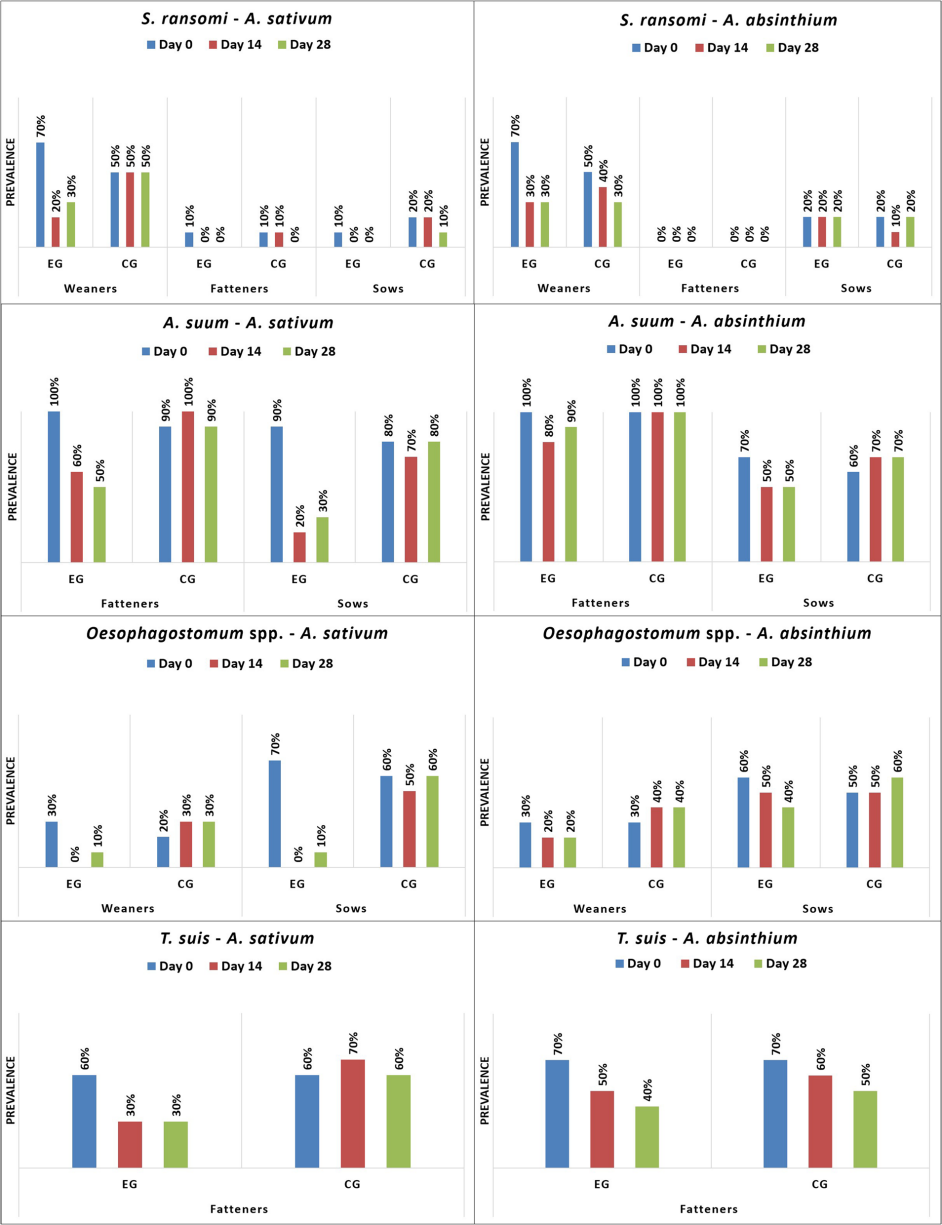
Prevalence (%) of investigated nematodes on farm 1 by age group (EG = experimental group; CG = control group)

**Fig. 3:**
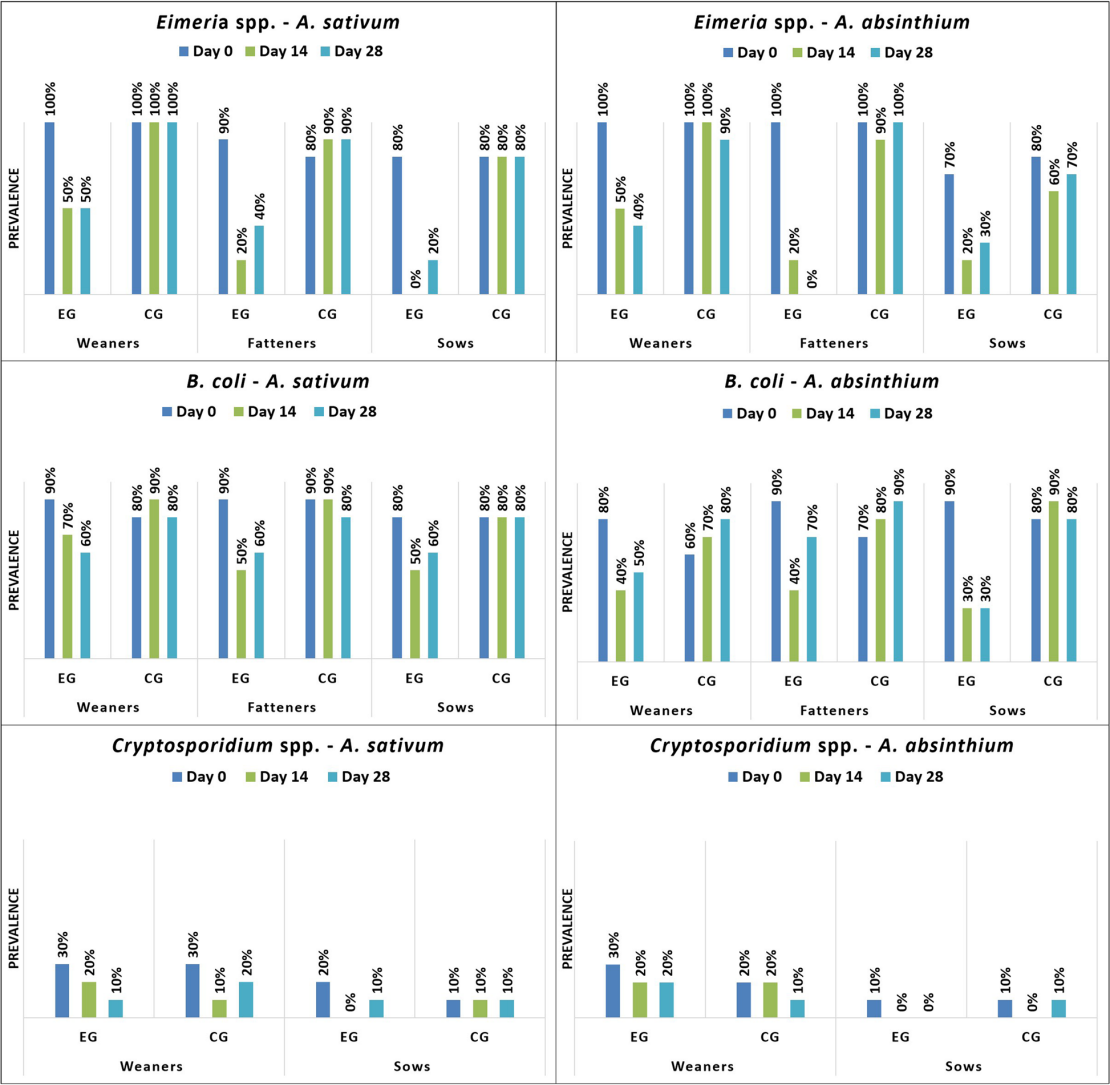
Prevalence (%) of investigated protozoa on farm 2 by age group (EG = experimental group; CG = control group)

**Fig. 4:**
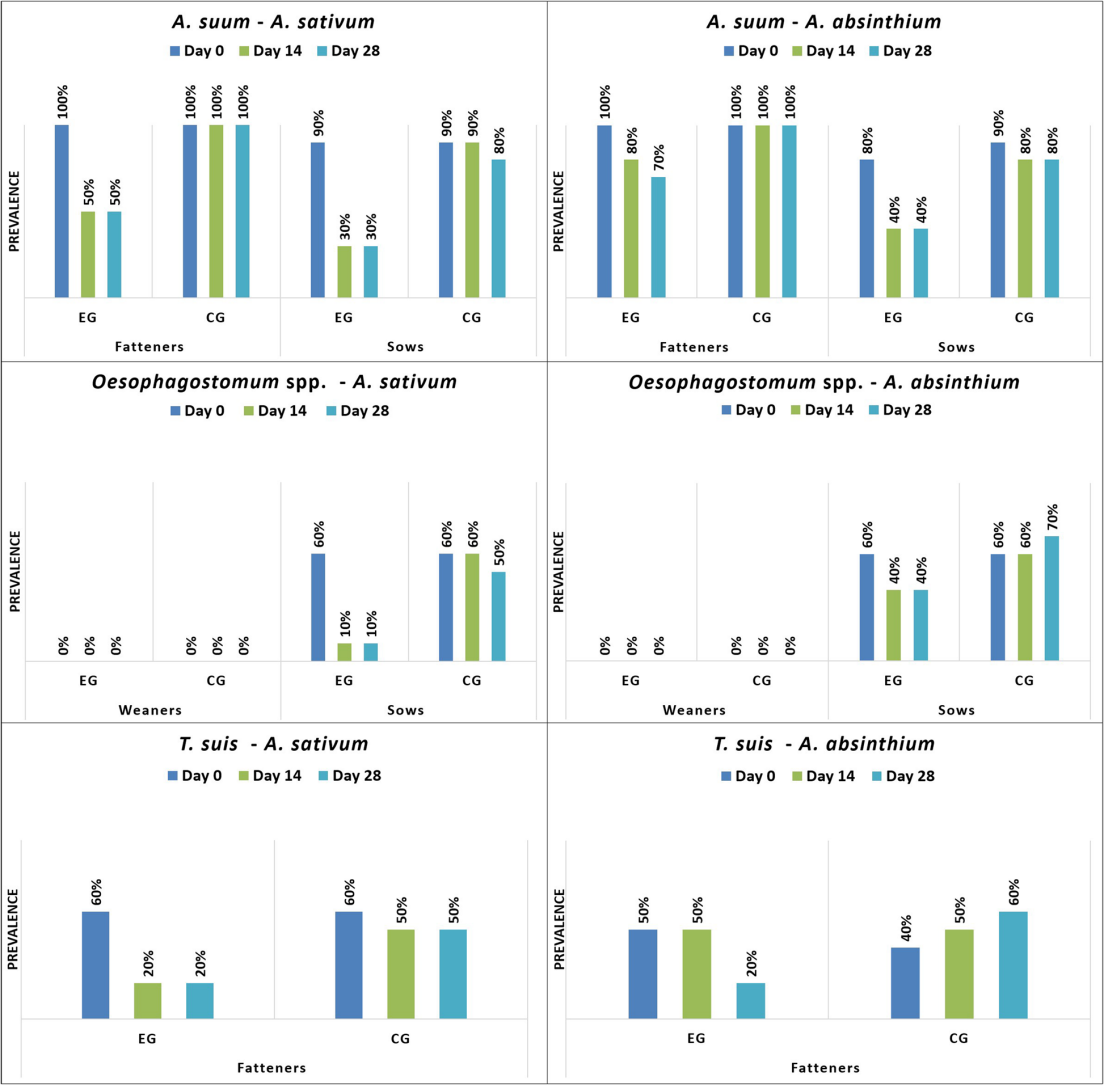
Prevalence (%) of investigated nematodes on farm 2 by age group (EG = experimental group; CG = control group)

*S. ransomi* was only found on farm 1 (F1), but it was present in all age categories. In weaners from farm 2 (F2), only *Eimeria* spp., *B. coli* and *Cryptosporidium* spp. were diagnosed, while on F1, *Oesophagostomum* spp. was additionally found. In fatteners, *Eimeria* spp., *B. coli*, *T. suis*, and *A. suum* were identified on both farms. In sows from both farms, *Eimeria* spp., *B. coli*, *A. suum*, *Oesophagostomum* spp., and *Cryptosporidium* spp. were observed (Figs. 1, 2, 3, 4).

*A. sativum* demonstrated a strong antiparasitic (antiprotozoal, anthelmintic) activity against all diagnosed parasites and across all pig categories, except for *Cryptosporidium*, in the case of which both plants showed limited antiparasitic activity. While *A. absinthium* demonstrated a good antiparasitic efficacy against *Eimeria*, *B. coli*, *A. suum, Oesophagostomum*, and *T. suis*, it had no noticeable effect against *S. ransomi*. In both plants, the strongest antiparasitic activity against all parasites was demonstrated on day 14, with a decrease of the therapeutic effect following day 28 (Figs. 1, 2, 3, 4; Table 1, 2, 3).

**Table 1:** Antiparasitic effects of *A. sativum* and *A. absinthium* in farm 1 by age group

Parasite	Day	*A. sativum*			*A. absinthium*		
		**EG**	**CG**	***P***	**EG**	**CG**	***P***
**Weaners**							
*Eimeria* spp.	0	100 [0–550]	0 [0–50]	0.4727^#^	2600 [2100–3150]	2400 [1950–2950]	0.7337^#^
	14	0 [0–0]	200 [0–400]	0.0963^#^	0 [0–150]	2400 [1500–2950]	0.0012^#^
	28	0 [0–0]	400 [0–750]	0.0539^#^	0 [0–600]	2400 [1850–2600]	0.0002^#^
	***P***	0.0907*	0.4794*		0.0004*^A^	0.8825*	
*B. coli*	0	200 [0–550]	0 [0–350]	0.4963^#^	500 [200–900]	500 [250–750]	0.9699^#^
	14	0 [0–0]	200 [0–750]	0.1736^#^	0 [0–0]	600 [400–800]	0.0013^#^
	28	0 [0–150]	300 [0–600]	0.1306^#^	0 [0–150]	600 [250–750]	0.0102^#^
	***P***	0.0839*	0.7979*		0.0128*^B^	0.8669*	
*Oesophagostomum* spp.	0	0 [0–150]	0 [0–0]	0.7337^#^	0 [0–38]	0 [0–38]	0.9699^#^
	14	0 [0–0]	0 [0–38]	0.2730^#^	0 [0–0]	0 [0–163]	0.4963^#^
	28	0 [0–0]	0 [0–150]	0.4057^#^	0 [0–0]	0 [0–163]	0.5205^#^
	***P***	0.1738*	0.8007*		0.9487*	0.8669*	
S. *ransomi*	0	300 [50–550]	100 [0–400]	0.4963^#^	200 [13–400]	100 [0–350]	0.4963^#^
	14	0 [0–0]	100 [0–350]	0.1620^#^	0 [0–38]	0 [0–200]	0.5967^#^
	28	0 [0–150]	200 [0–400]	0.1988^#^	0 [0–150]	0 [0–300]	0.8501^#^
	***P***	0.0024*^a^	0.6065*		0.0204*	0.8187*	
**Fatteners**							
*Eimeria* spp.	0	0 [0–400]	0 [0–38]	0.6501^#^	2200 [1650–2400]	900 [450–2150]	0.0588^#^
	14	0 [0–0]	0 [0–500]	0.3643^#^	0 [0–450]	2100 [1800–2550]	0.0012^#^
	28	0 [0–0]	0 [0–350]	0.4274^#^	0 [0–0]	2000 [1400–2600]	0.0046^#^
	***P***	0.2466*	0.9608*		0.0001*^C^	0.6412*	
*B. coli*	0	700 [250–1550]	600 [250–600]	0.3847^#^	800 [500–1100]	600 [450–1000]	0.8206^#^
	14	200 [50–400]	700 [600–950]	0.0494^#^	0 [0–150]	700 [450–950]	0.0102^#^
	28	200 [50–550]	700 [250–1150]	0.1306^#^	100 [0–550]	900 [450–1200]	0.0211^#^
	***P***	0.3368*	0.5899*		0.0015*^D^	0.8276*	
*A. suum*	0	2300 [550–8000]	3400 [2850–3750]	0.8798^#^	2800 [1200–11000]	5650 [3950–7500]	0.5708^#^
	14	600 [0–950]	3500 [3200–3750]	0.0002^#^	1400 [700–1950]	6500 [3850–8100]	0.0003^#^
	28	200 [0–750]	3500 [3100–4100]	0.0015^#^	1400 [1200–1900]	5400 [3750–7150]	0.0002^#^
	***P***	0.0134*^b^	0.7841*		0.2725*	0.7891*	
*T. suis*	0	300 [0–550]	200 [0–550]	0.8501^#^	400 [50–750]	300 [50–550]	0.6232^#^
	14	0 [0–38]	400 [100–600]	0.0312^#^	25 [0–350]	400 [0–550]	0.3447^#^
	28	0 [0–150]	400 [0–600]	0.1041^#^	0 [0–350]	200 [0–550]	0.3847^#^
	***P***	0.1298*	0.8233*		0.1000*	0.9156*	
**Sows**							
*Eimeria* spp.	0	600 [200–950]	700 [300–950]	0.7337^#^	700 [450–1150]	500 [100–750]	0.1620^#^
	14	0 [0–0]	700 [300–950]	0.0008^#^	0 [0–150]	325 [0–950]	0.1306^#^
	28	0 [0–0]	325 [13–950]	0.0191^#^	0 [0–150]	700 [50–950]	0.0257^#^
	***P***	0.0008*^c^	0.3679*		0.0036*^E^	0.5811*	
*B. coli*	0	200 [13–750]	400 [13–750]	0.9397^#^	800 [350–1000]	800 [300–1150]	0.8501^#^
	14	0 [0–38]	500 [50–750]	0.0257^#^	0 [0–350]	800 [500–1100]	0.0113^#^
	28	0 [0–200]	600 [250–950]	0.0140^#^	200 [0–600]	700 [450–1150]	0.0312^#^
	***P***	0.1225*	0.8669*		0.0116*	0.3147*	
*A. suum*	0	700 [200–1200]	200 [88–1200]	0.5708^#^	400 [50–600]	300 [0–550]	0.5967^#^
	14	0 [0–0]	200 [50–1200]	0.0233^#^	100 [0–350]	400 [50–750]	0.1620^#^
	28	0 [0–150]	400 [400–600]	0.0058^#^	100 [0–550]	500 [100–600]	0.2899^#^
	***P***	0.0004*^d^	0.8669*		0.4493*	0.8035*	
*Oesophagostomum* spp.	0	200 [13–350]	125 [0–350]	0.7913^#^	300 [0–550]	100 [0–350]	0.7055^#^
	14	0 [0–0]	100 [0–200]	0.0640^#^	25 [0–350]	300 [0–600]	0.4274^#^
	28	0 [0–0]	300 [0–700]	0.0413^#^	0 [0–350]	400 [0–950]	0.2123^#^
	***P***	0.0046*^e^	0.9487*		0.5719*	0.4124*	

*EG* Experimental group, *CG* Control group

Results are expressed as median [Q1-Q3], where Q1 is the 25th percentile and Q3 is the 75th percentile;

*p* are the estimated probabilities associated to *Friedman’s or ^#^ Mann–Whitney test;

Wilcoxon test: ^a^ 0.0180 day 0 vs. day 14, 0.0277 day 0 vs. day 28, 0.0180 day 14 vs. day 28; ^b^ 0.0218 day 0 vs. day 14, 0.0125 day 0 vs. day 28, 0.0218 day 14 vs. day 28; ^c^ 0.0117 day 0 vs. day 14, 0.0180 day 0 vs. day 28, 0.0117 day 14 vs. day 28; ^d^ 0.0077 day 0 vs. day 14, 0.0117 day 0 vs. day 28, 0.0077 day 14 vs. day 28; ^e^ 0.0180 day 0 vs. day 14, 0.04232 day 0 vs. day 28, 0.0180 day 14 vs. day 28;

^A^ 0.0077 day 0 vs. day 14, 0.0051 day 0 vs. day 28, 0.0077 day 14 vs. day 28; ^B^ 0.0209 day 0 vs. day 14, 0.0251 day 0 vs. day 28, 0.0209 day 14 vs. day 28; ^C^ 0.0051 day 0 vs. day 14, 0.0051 day 0 vs. day 28, 0.0051 day 14 vs. day 28; ^D^ 0.0117 day 0 vs. day 14, 0.0180 day 0 vs. day 28, 0.0117 day 14 vs. day 28; ^E^ 0.0117 day 0 vs. day 14, 0.0129 day 0 vs. day 28, 0.0117 day 14 vs. day 28

**Table 2:** Antiparasitic effects of *A. sativum* and *A. absinthium* in farm 2 by age group

Parasite	Day	*A. sativum*			*A. absinthium*		
		**EG**	**CG**	***P***	**EG**	**CG**	***P***
**Weaners**							
*Eimeria* spp.	0	2400 [2050–2550]	2000 [1500–2400]	0.2899^#^	5900 [4750–8450]	6900 [4850–9350]	0.7913^#^
	14	100 [0–350]	2200 [1700–2400]	0.0002^#^	400 [0–950]	6700 [5500–8450]	0.0002^#^
	28	25 [0–350]	2500 [2400–2600]	0.0002^#^	0 [0–800]	6400 [5450–6950]	0.0013^#^
	***P***	0.0003*^a^	0.4786*		0.0002*^g^	0.7103*	
*B. coli*	0	1000 [450–1350]	500 [250–1350]	0.5967^#^	400 [50–750]	125 [0–350]	0.1859^#^
	14	200 [13–350]	1000 [650–1400]	0.0036^#^	0 [0–50]	400 [100–550]	0.0312^#^
	28	125 [0–350]	800 [600–1200]	0.0140^#^	25 [0–200]	600 [200–750]	0.0312^#^
	***P***	0.1211*	0.6246*		0.0137*	0.0312*	
**Fatteners**							
*Eimeria* spp*.*	0	500 [250–1100]	300 [200–900]	0.4497^#^	2200 [1650–2400]	1700 [750–3000]	0.6776^#^
	14	0 [0–0]	1000 [600–1700]	0.0012^#^	0 [0–0]	1900 [1500–2550]	0.0025^#^
	28	0 [0–163]	1500 [1250–1600]	0.0013^#^	0 [0–0]	2000 [1400–2600]	0.0002^#^
	***P***	0.0018*^b^	0.0458*		0.0006*^h^	0.9718*	
*B. coli*	0	800 [450–1100]	600 [450–950]	0.6501^#^	800 [250–1100]	400 [50–600]	0.1620^#^
	14	25 [0–163]	800 [450–1200]	0.0036^#^	0 [0–550]	500 [250–800]	0.1212^#^
	28	300 [0–400]	1300 [400–1550]	0.0343^#^	400 [50–750]	1400 [1200–1750]	0.0025^#^
	***P***	0.05721*	0.30287*		0.32465*	0.01882*	
*A. suum*	0	3200 [1300–11000]	2800 [1050–11000]	0.7055^#^	2800 [1050–11000]	6600 [3750–7500]	0.5708^#^
	14	400 [0–1150]	6100 [3450–7950]	0.0002^#^	1700 [1250–2300]	6500 [3850–8100]	0.0010^#^
	28	100 [0–550]	4100 [1800–8050]	0.0006^#^	1200 [100–1900]	3200 [1300–8000]	0.0312^#^
	***P***	0.0008*^c^	0.8890*		0.5669*	0.5004*	
*T. suis*	0	200 [0–350]	200 [0–400]	0.9699^#^	25 [0–350]	0 [0–200]	0.6776^#^
	14	0 [0–0]	25 [0–550]	0.1736^#^	300 [0–1200]	200 [0–400]	0.4057^#^
	28	0 [0–0]	100 [0–350]	0.1859^#^	0 [0–0]	900 [0–1350]	0.0539^#^
	***P***	0.0316*	0.6483*		0.2890*	0.1396*	
**Sows**							
*Eimeria* spp.	0	400 [88–600]	400 [250–600]	0.8798^#^	600 [50–800]	400 [88–600]	0.5454^#^
	14	0 [0–0]	600 [400–950]	0.0028^#^	0 [0–0]	700 [0–1000]	0.0539^#^
	28	0 [0–0]	600 [600–800]	0.0046^#^	0 [0–38]	700 [150–950]	0.0233^#^
	***P***	0.0019*^d^	0.4124*		0.0076*^i^	0.3282*	
*B. coli*	0	700 [600–1350]	700 [200–950]	0.4497^#^	700 [600–1350]	400 [200–800]	0.1509^#^
	14	25 [0–350]	800 [250–800]	0.0312^#^	0 [0–38]	800 [450–1100]	0.0017^#^
	28	125 [0–400]	700 [450–950]	0.0312^#^	0 [0–38]	700 [450–1150]	0.0065^#^
	***P***	0.0266*	0.4223*		0.0005*^j^	0.3679*	
*A. suum*	0	300 [88–600]	500 [200–600]	0.7055^#^	500 [138–750]	300 [88–600]	0.5708^#^
	14	0 [0–38]	500 [250–800]	0.0019^#^	0 [0–163]	500 [250–800]	0.0140^#^
	28	0 [0–38]	600 [400–750]	0.0073^#^	0 [0–163]	600 [400–750]	0.0091^#^
	***P***	0.0014*^e^	0.4124*		0.0753*	0.3679*	
*Oesophagostomum* spp.	0	125 [0–350]	200 [0–350]	0.7913^#^	500 [0–950]	125 [0–350]	0.4057^#^
	14	0 [0–0]	225 [0–600]	0.0413^#^	0 [0–400]	400 [0–750]	0.2899^#^
	28	0 [0–0]	100 [0–550]	0.1041^#^	0 [0–550]	600 [100–1000]	0.1041^#^
	***P***	0.0032*^f^	0.5811*		0.3679*	0.2564*	

*EG* Experimental group, *CG* Control group

Results are expressed as median [Q1-Q3], where Q1 is the 25th percentile and Q3 is the 75th percentile; p are the estimated probabilities associated to *Friedman’s or ^#^ Mann–Whitney test;

Wilcoxon test:^a^ 0.0051 day 0 vs. day 14, 0.0051 day 0 vs. day 28, 0.0051 day 14 vs. day 28; ^b^ 0.0077 day 0 vs. day 14, 0.0152 day 0 vs. day 28, 0.0077 day 14 vs. day 28; ^c^ 0.0077 day 0 vs. day 14, 0.0051 day 0 vs. day 28, 0.0077 day 14 vs. day 28; ^d^ 0.0117 day 0 vs. day 14, 0.0129 day 0 vs. day 28, 0.0117 day 14 vs. day 28; ^e^ 0.0166 day 0 vs. day 14, 0.0077 day 0 vs. day 28, 0.01661 day 14 vs. day 28; ^f^ 0.0277 day 0 vs. day 14, 0.0277 day 0 vs. day 28, 0.0277 day 14 vs. day 28;

^g^ 0.0051 day 0 vs. day 14, 0.0051 day 0 vs. day 28, 0.0051 day 14 vs. day 28; ^h^ 0.01086 day 0 vs. day 14, 0.0051 day 0 vs. day 28, 0.0109 day 14 vs. day 28; ^i^ 0.0173 day 0 vs. day 14, 0.0173 day 0 vs. day 28, 0.0173 day 14 vs. day 28; ^j^ 0.0077 day 0 vs. day 14, 0.0077 day 0 vs. day 28, 0.0077 day 14 vs. day 28

**Table 3:** Percentage of faecal egg/oocyst/cyst count reduction (%) recorded on days 14, and 28 post-treatment in F1 and F2 farms (using FECR formula)

**Parasite**	***A. sativum*** (14)						***A. sativum*** (28)					
	**Weaners**		**Fatteners**		**Sows**		**Weaners**		**Fatteners**		**Sows**	
	**F1**	**F2**	**F1**	**F2**	**F1**	**F2**	**F1**	**F2**	**F1**	**F2**	**F1**	**F2**
*Eimeria* spp*.*	76.7	82.1	62.1	79.6	100	100	88.1	84.6	20.0	84.1	78.9	83.5
*B. coli*	59.8	74.2	76.1	75.1	82.3	66.3	47.9	72.3	66.7	69.8	55.8	67.8
*A. suum*	-	-	82.3	79.8	87.6	72.1	-	-	84.7	86.3	68.2	62.8
*T. suis*	-	-	66.7	76.6	-	-	-	-	63.9	54.1	-	-
*Oesophagostomum* spp.	100	-	-	-	100	87.5	88.7	-	-	-	67.3	45.8
*S. ransomi*	64.4	-	100	-	100	-	57.3	-	100	-	100	-
**Parasite**	***A. absinthium*** ** (14)**						***A. absinthium*** ** (28)**					
	**Weaners**		**Fatteners**		**Sows**		**Weaners**		**Fatteners**		**Sows**	
	**F1**	**F2**	**F1**	**F2**	**F1**	**F2**	**F1**	**F2**	**F1**	**F2**	**F1**	**F2**
*Eimeria* spp*.*	74.2	84.0	71.8	33.1	65.8	92.4	71.5	84.9	85.1	100	56.3	89.8
*B. coli*	72.1	88.4	60.3	37.7	58.7	88.0	63.3	80.6	46.9	71.9	31.6	85.1
*A. suum*	-	-	71.3	64.9	44.7	80.5	-	-	70.4	64.3	30.2	78.6
*T. suis*	-	-	50.4	39.5	-	-	-	-	49.9	79.2	-	**-**
*Oesophagostomum* spp.	33.2	-	-	-	49.5	63.1	25.1	-	-	-	43.8	66.7
*S. ransomi*	36.2	-	-	-	44.4	-	31.3	-	-	-	69.1	**-**

“-” = was not diagnosed

Both control and experimental groups were homogeneous at the beginning of the experimental period, before the treatment (Table 1 and 2). For all of *Eimeria* spp., *B. coli*, *A. suum*, *T. suis*, *Oesophagostomum* spp., and *S. ransomi,* the differences between experimental and control groups (EG 14 / CG 14, EG 28 / CG 28) on one hand, and between experimental groups (EG 0 / EG 14 / EG 28) on the other hand, were statistically significant for each farm, plant, and age category. The *A. sativum* group had statistically significant values (SSVs) for *Eimeria* spp. in sows from F1 and in all age groups from F2, for *B. coli* in sows from F1 and in all age groups from F2, for *A. suum* in fatteners and sows from both farms, for *S. ransomi* in weaners from F1, and for *Oesophagostomum* spp. in sows from both farms (Table 1 and 2). The *A. absinthium* group showed SSVs for *Eimeria* spp. and *B. coli* in all age groups from both farms, and for *A. suum* in sows from F2 and in fatteners from both farms (Table 1 and 2).

The therapeutic efficacy (reduction %) of *A. sativum* (AS) and *A. absinthium* (AA) against diagnosed parasites, in all age groups ranged as follows: AS = 20–100%, AA = 33.1–100% for *Eimeria* spp., AS = 47.9–82.3%, AA = 31.6–88.4% for *B. coli*, AS = 62.8–87.6%, AA = 30.2–80.5% for *A. suum,* AS = 54.1–76.6%, AA = 39.5–79.2%, for *T. suis*, AS = 45.8–100%, AA = 25.1–66.7% for *Oesophagostomum* spp., and AS = 57.3–100%, AA = 31.3–69.1% for *S. ransomi* (Table 3). Due to a lack of quantitative sensitivity of usual coproparasitological methods for *Cryptosporidium* in terms of the oocysts numbers (intensity)*,* the infection prevalence was the only indicator provided.

## Discussion

In this experiment, the evaluation of the antiparasitic efficacy of two plants from the Romanian flora (*A. sativum* and *A. absinthium*) was carried out successfully on two low-input farms. Despite the lack of scientific data on the effect of the abovementioned plants on swine parasites, we extrapolated our results by comparing them to studies on humans [19], and domestic species [13]. Overall, all three age groups (weaners, fatteners and sows) had similar co-infections with protozoa and nematodes. The efficacy of plants was estimated by calculating the difference between the parasitic intensity before and after the garlic and wormwood therapy. In most of the cases, the intensity was highly influenced along with the prevalence, while in others, the prevalence remained unchanged in spite of decreasing the intensity of the infection.

Previous reports mention a daily *A. sativum* dose ranging from a minimum of 30 mg to a maximum of 1052 mg/kg bw [31]. In the present study, the selected dose of 180 mg garlic powder/kg bw/day was administered to infected swine, for ten consecutive days. This dose was deemed safe, avoiding any potential adverse effects, i.e. lack of appetite, weight loss, liver injury, pulmonary oedema, inhibition of spermatogenesis, partial paralysis, allergy, anaemia and death, previously attributed to compounds like diallyl disulfide, allylpropyl sulfide, and allicin [20, 32]. For *A. absinthium*, a dosage of 90 mg/kg bw/day for 10 days was used under the same considerations as for garlic. There was no available information on the therapeutic dose of wormwood used in pigs, therefore the choice of tested doses was based on studies on other species. These included rats (300 mg/kg/day), goats (2000 mg/kg, single dose) and humans (2000–3000 mg/individual/day) [33–35]. Long-term administration of *A. absinthium* can cause neurotoxicity due to the presence of thujone and trans-sabinyl acetate. Additionally, adverse side effects, at least in humans, may include gastrointestinal disorders, brain injury, vertigo, insomnia, restlessness, urine retention, seizures, tremors, and even death [14, 22]. In our study, no adverse reactions were observed, if the therapeutic doses were respected.

The chemical composition of the two Romanian medicinal plants used in the present study resembles those from previous reports, only differing in concentration. Biological compounds, such as polyphenols, tocopherols, flavonoids, sesquiterpene lactones, and sulfoxide, have demonstrated strong antiprotozoal and anthelmintic properties, both in vitro and in vivo. Therefore, they could be used as substitutes for classic antiparasitics [4, 11, 12, 15, 22, 36].

*Eimeria* spp. was diagnosed in weaners, fatteners and sows, in both farms, with high prevalence. *A. sativum* and *A. absinthium* powders demonstrated a strong anticoccidial activity (reducing oocyst excretion) in all age groups, in both farms, with wormwood (33.1%-100%) displaying superior efficacy to that of the garlic (20%-100%) (Table 3). Although eimeriosis is of low pathogenicity in swine compared to other species (birds, rabbits, ruminants) in which the disease is more clinically relevant, the efficacy provided by garlic and wormwood powders in our survey is comparable with the results obtained in respective studies. The propylene glycol extract from *A. sativum* and *Thymus serpyllum* reduced duodenal lesions, increasing the feed conversion ratio and oocysts output, as well as reducing the weight gain in broilers compared to control groups, treated with amprolium [37]. Abu-Akkada et al. [38] showed that oral administration of crude garlic in a daily dosage of 500 mg/kg bw for five consecutive days limited the adverse impacts of hepatic coccidiosis in rabbits, resulting in improved body weight gain and lowered numbers of oocysts. Alcoholic garlic extract was proved to possess a remarkable anticoccidial effect against rabbit intestinal coccidiosis, both in vitro and in vivo, and could be used as a natural feed additive for both prophylaxis and treatment of coccidiosis, thus minimizing the economic losses caused by the parasite [39]. Sidiropoulou et al. [40] showed that supplementation of oregano and garlic essential oils had a significant anticoccidial effect in vitro, along with promoting growth in broilers, reared in the absence of anticoccidial drugs. Artemisinin is a sesquiterpene lactone isolated from *Artemisia absinthium*, with an unclear action mechanism, still used in controlling poultry coccidiosis [27]. Several studies have demonstrated that *A. absinthium (*essential oil, leaf powder), used as an additive in chickens, exhibited anticoccidial activity by lowering the severity of diarrhoea, as well as reducing oocyst excretion, improving feed conversion efficiency, and strengthening the immune system [30, 41, 42]. Therapy with *A. absinthium* extracts, using a dose of 1–3 mg/kg, was able to reduce the severity of *Eimeria* infection, thus decreasing the number of oocysts per gram of faeces in chickens infected with *Eimeria tenella* [43]. Popović et al. [44] also noticed that wormwood supplementation (100 g/kg) in the diet of rabbits had an anticoccidial effect, along with a positive influence on growth performance, as well as on antioxidative systems. The positive results obtained in this experiment are highly valuable for the prevention and control of porcine coccidiosis, especially in organic and low-input farming systems, particularly because in vivo studies regarding the efficacy of wormwood and garlic on *Eimeria* species in pigs, are lacking.

Cryptosporidiosis is an emerging zoonotic protozoan parasite that poses a global challenge in both veterinary and human medicine [45]. The findings revealed its existence, while the usual coproparasitological tehniques lacked the ability to detect the average intensity, due to the absence of quantitative sensitivity in oocyst counting. In the present study, *Cryptosporidium* spp. was only identified in weaners and sows, which were considered to serve as a permanent source of infection. Both plants exhibited limited efficacy against *Cryptosporidium,* causing a decrease in prevalence (Fig. 1 and Fig. 3). In *Cryptosporidium* infected mice, treatment with raw garlic juice (50 mg/kg, for seven days) also reduced shedding of *Cryptosporidium* oocysts [45]. In humans, therapy with allicin against *Cryptosporidium parvum* and *Cryptosporidium hominis* was successful, indicating a lethal effect on *Cryptosporidium*, while improving patient immune function [46, 47]. Gaafar et al. [48] performed an in vivo experiment, administering aqueous garlic extract on immunosuppressed mice, and observed a destruction of *Cryptosporidium* oocysts. The use of artemisin against *C. parvum* infection showed limited effectiveness, causing a minor decrease in average oocyst numbers, when compared to macrolides [49], similar to the results obtained in the present study in pigs. *Artemisia* ethanolic extracts were used as a remedy in mouse cryptosporidiosis and the results revealed significantly reduced oocyst shedding, while the symptoms disappeared in the treated groups [50]. *A. absinthium* essential oil (2 mg/ml) was able to destroy *Cryptosporidium baileyi* and *Cryptosporidium galli* oocysts, in vitro [51]. Considering that the selected age groups were only carriers of the parasite, the practical importance of the effect of garlic and wormwood on cryptosporidiosis might be limited. *Cryptosporidium* clinically affects only piglets during neonatal period. Therefore, the positive results obtained by both plants, might not have a significant impact in this frame.

*Balantioides coli* (former *Balantidium coli*) was diagnosed in all age groups, in both farms. *A. sativum* (47.9%-82.3%) and *A. absinthium* (31.6%-88.4%) were both effective against balantidiasis. However, garlic had a more pronounced antiparasitic activity (Table 3). No studies were found on the antiparasitic activity of garlic and wormwood against balantidiasis, although these plants have been proven efficient against numerous protozoa, such as: *Toxoplasma* spp.*,* *Plasmodium* spp.*,* *Entamoeba histolytica, Leishmania* spp.*, Trypanosoma cruzi*, *Trichomonas vaginalis, Giardia lamblia* [16, 22, 23, 46, 47, 52–57]. An in vivo study revealed that aqueous garlic extracts (200 mg/kg bw) had the highest antitrichomonal effect, thus shortening the duration of the treatment of pigeons from seven to five days [58]. Therefore, it may be concluded that the present report is at the forefront of evaluating the antiparasitic activity of these plants against *B. coli.*

*Ascaris suum*, another important parasite, was identified in fatteners and sows. Both *A. sativum* (62.8%-87.6%) and *A. absinthium* (30.2%-80.5%) demonstrated a strong anthelmintic activity against *A. suum* and may represent an alternative therapy to classic antiparasitic drugs (Table 3). The antiparasitic efficacy of both plants used in the present experiment has been confirmed by previous literature reports. Akoh et al. [19] indicated that the ethanolic garlic extract was used against *Ascaris lumbricoides* in humans with egg counts decreasing by 80.73% (200 mg/kg bw of garlic extract), 84.26% (400 mg/kg bw) and by 91.78% (800 mg/kg bw), respectively. According to the studies conducted by Raza et al. [13], *A. sativum* also had an anthelmintic activity against *A. galli* in chickens, attributed to the presence of allicin. However, this effects was not as pronounced as that of flubendazole. In an in vitro study, ethanolic and aqueous extracts of *A. sativum* inhibited hatching, by killing 100% of the larvae of *Ancilostoma caninum* and *Toxocara canis*, at concentrations between 1.25 and 10 mg/ml [32]. *A. absinthium* extract administrated orally to cats at a dose between 300–600 mg/kg bw showed a good anthelmintic activity [59]. Wormwood methanolic extracts demonstrated a strong in vitro inhibitory effect on the embryonation rate of *A. galli* eggs [60]. Several reports highlighted the anthelmintic activity of *A. absinthium* against *A. suum* and *A. galli* [61–63]. Since *A. suum* infection has a negative impact on the health and welfare of pigs, the excellent results obtained in this study are highly beneficial, offering a promising alternative for the treatment of this parasitosis mainly on organic and low-input farms.

*Oesophagostomum* spp. was the only strongyle diagnosed. This parasite was identified in both farms, in weaners and sows. Our results showed that *A. sativum* (45.8%-100%) was very effective against *Oesophagostomum*, while *A. absinthium* (25.1%-66.7%) expressed a weak antiparasitic activity (Table 3)*.* In an in vivo study, the effect of aqueous and alcoholic extracts of *A. sativum* on gastrointestinal endoparasites of sheep (*Haemonchus*, *Cooperia*, *Trichostrongylus*) was evaluated, inducing a reduction in EPG in treated groups [13, 64]. *A. sativum* showed also a good efficacy against nematodes of cattle using a dose of 100 mg/kg bw for 28 days [65]. A good efficacy in reduction of strongyle eggs in goats treated with raw garlic juice with different concentrations (between 20–80%) was obtained by Masamha et al. [66], the effect increasing with higher concentrations. Oral administration of garlic formulations had no effect on the egg shedding of intestinal strongyles in horses [67]. In the present study in swine, *A. absinthium* exhibited only poor effects on nematodes compared to previous findings revealing the anthelmintic effectiveness of *A. absinthium* against small ruminants nematodes (*Haemonchus contortus, Teladorsagia circumcincta*, and *Chabertia ovina*), both in vivo and in vitro [62, 68–70].

*Strongyloides ransomi* mainly infects young pigs, causing only sporadically a clinical disease [71]. In this study, it was only diagnosed in farm 1, in all age groups. The presented results demonstrated that garlic (57.3%-100%) was strongly effective against *S. ransomi*, while wormwood (31.3%-69.1%) had only a mild effect (Table 3). Fawzi and Elsohaby [72] investigated the role of allicin in treatment of gastrointestinal nematodes (*Trichostrongylus*, *Cooperia*, and *Strongyloides*) in cattle and demonstrated that allicin had an anthelmintic action comparable to that of albendazol. Suttun et al. [16] showed that *A. sativum* is effective against *Strongyloides* infection in donkeys. Consistently, Ahmed et al. [73] evaluated in vivo effects of ethanolic *A. sativum* extract (100 mg/kg bw) and demonstrated a good anthelmintic effect against sheep gastrointestinal nematodes, including *Strongyloides*. To our best knowledge, no other reports on the antiparasitic activity of *A. absinthium* against strongyloidosis were published, therefore, our results could provide a valuable resource for future studies in farm species.

Lastly, *Trichuris suis* was diagnosed. The whipworm was identified in both farms, affecting only fatteners, thus supporting the life cycle of this parasite. Our study revealed that both plants, *A. sativum* (54.1%-76.6%) and *A. absinthium* (39.5%-79.2%), were effective against *T. suis* (Table 3). Consistently, aqueous garlic extracts showed a good activity against *Trichuris muris* [74]. *A. sativum,* extracted with various solvents and used in different experimental models, showed a good anthelmintic activity against other nematodes, such as: *Heterakis gallinarum*, *Angiostrongylus cantonensis*, and *Trichinella spiralis* [53, 75, 76]. Mirza et al. [28] demonstrated the antiparasitic efficacy of terpenes, chemical compounds found in *A. absinthium,* against *T. muris* and *Ancylostoma ceylanicum.* Similarly, wormwood had a potent anthelmintic activity against *T. spiralis* and *H. gallinarum* [33, 77].

In summary, the aforementioned studies corroborated with our current study demonstrated that *A. sativum* and *A. absinthium* could be prospective sources for the development of new and potent antiparasitic herbal remedies, for both humans and animals. Plant powders, along with essential oils, aqueous and alcoholic extracts, have been found to exhibit strong antiprotozoal and anthelminthic effects. Allicin (garlic) and artemisinin (wormwood) are two of the most important bioactive molecules responsible for the antiparasitic activity. This study identified a co-infection parasitic status, including protozoa and helminths on all studied farms. On one of the farms (F1) a supplementary parasite, *S. ransomi,* was present. Nevertheless, the effects of both plants were similar on both farms.

The results obtained from our research have practical implications for organic livestock systems and the welfare and health of pigs. Moreover, considering the zoonotic potential of several of the identified parasites (*A. suum*, *Cryptosporidium*, and *B. coli*), the dissemination of such studies is of the utmost importance.

## Conclusion

The current study demonstrated that administering powdered *A. sativum* bulbs and *A. absinthium* aerial parts at doses of 180 mg/kg/day and 90 mg/kg/day, respectively, for ten consecutive days, may be be effective against digestive parasites in swine.

The findings of the present study revealed that *A. sativum* and *A. absinthium* have the potential of treating gastrointestinal parasitoses in swine. The curative efficacy may be attributed to the presence of polyphenols, sterols, tocopherols, flavonoids, sesquiterpene lactones, and sulfoxide, which exhibit antiparasitic activity. Therefore, *A. sativum* and *A. absinthium* have satisfactory antiparasitic (antiprotozoal and anthelmintic) potential and may still be of value as part of an integrated approach, specifically designed to achieve sustainable parasite control in low-input swine production systems.

The absence of toxicity observed for garlic and wormwood, coupled with our study results, leads us to propose that these medicinal plants could serve as a foundation for developing a novel line of antiparasitic herbal medication.

Further studies on the standardization of dosage and identification of precise action mechanisms of these plants against the gastrointestinal parasites are thereby demanded. The findings from the present study contribute to the field of plant antiparasitic therapies allowing sustainable, effective and safe alternative development, along with preventive care through additives based on these compounds.

## Methods

### Ethics statement and ontologies

Before and during the experiment, the behaviour and clinical condition of the pigs was monitored. The described experiment complied with national (Law No. 43 of 2014) and European law (EU Directive No. 63 of 2010) with respect to animal experimentation and care of animals under study. The ontologies related to medicinal plants, chemical compounds, diseases, and pathogens utilised in the experimental protocol were detailed in Additional file 1.

### Ethics approval and consent to participate

The study was conducted in accordance with the Declaration of Helsinki and approved by the Ethics Committee of the University of Agricultural Sciences and Veterinary Medicine of Cluj-Napoca, Romania (Permit no. 231/02.11.2020). The current experiment was conducted on two farms, and informed consent was obtained from the owners of both farms.

### Chemical analysis of A. sativum and A. absinthium

The aerial parts of *A. absinthium* (wormwood) and *A. sativum* (garlic) bulbs were processed into powder and were used for the chemical analysis. High performance liquid chromatography coupled with mass spectrometry (HPLC–MS) was used for the analysis of biologically active compounds present in the alcoholic plant extracts (Additional file 2). All the procedures were performed at the”Iuliu Haţieganu “ University of Medicine and Pharmacy Cluj-Napoca (Romania). The techniques, equipment and methods used for analysis of *A. sativum* L. and *A. absinthium* L. alcoholic extracts have been described in detail in a previous article [78].

### Swine husbandry

The faecal samples were obtained from two low-input farms (referred to as F1 and F2), both of which raised native Bazna and Mangalitza pig breeds. These breeds are renowned for their superior organoleptic characteristics of meat, resistance to diseases, minimal requirement for complex feed regimens, and suitability for free-range (low-input) farming practices**.** At the beginning of the experiment, in April 2021, F1 had a pig herd of 350 animals, while F2 had 320 animals. The farms were located in Northwestern Romania, in a hilly area characterized by pastures and forests with a specific temperate-continental climate [8, 79]. From an infrastructure standpoint, both farms were similar: the interior of the barns was divided into compartments, the flooring consisted of concrete with drainage holes incorporated, there were automatic waterers, and feed was supplied in troughs. Drinking water for the animals was provided from a local potable water source, which meets all necessary hygiene and quality standards for human use. The shelters underwent regular hygiene maintenance twice daily throughout the year. Additionally, prior to starting the experimental protocol, a rigorous mechanical cleaning and disinfection of barns and paddocks was carried out. The outdoor environment, comprising an earth paddock, was bordered by an electric fence for protection against predators and also wild boars. The pasture and enrichments were accessible to the animals at all times [8, 79].

### Experimental design

To ensure precise results, a pilot study was conducted on a limited number of animals before initiating the experimental protocol. It involved assessing various reports detailing doses of *A. sativum* and *A. absinthium*. Hence, the feeding behaviour of the pigs, potential toxic effects, and antiparasitic efficacy of the tested plants were carefully observed and monitored.

The plants were sourced from Romania through an authorized company (Plafar SA, Romania), which brings over 50 years of expertise in producing, processing, storing, and marketing medicinal and aromatic plants for human use throughout Romania. The identification of the plant material occurred in the laboratory of Plafar SA. The control samples of garlic and wormwood have been meticulously preserved and stored at both the Parasitology and Parasitic Diseases Department at the University of Agricultural Sciences and Veterinary Medicine of Cluj-Napoca and the Department of Pharmaceutical Botany within the”Iuliu Haţieganu “ University of Medicine and Pharmacy of Cluj-Napoca.

The aerial parts of *A. absinthium* and the bulbs of *A. sativum* were ground, resulting in a feed containing either garlic or wormwood. Each type was then mixed with cereal flour. A total of 240 pigs were included in the study, with 120 pigs allocated to each plant based experiment variant, on both farms. Three age groups were defined in each of the studied pig herds, namely weaners, aged between 10 and 12 weeks, weighing 13–15 kg, fatteners, between 5 and 6 months, weighing 55–60 kg, and sows, aged from 1 to 4 years, with a body weight of 155 to 160 kg. On each farm and for each plant, three control groups (10 weaners, 10 fatteners, and 10 sows) and 3 experimental groups (10 weaners, 10 fatteners, and 10 sows) were defined. In F1, the study was conducted on pigs belonging to the Bazna breed, while in F2, Mangalitza pigs were used [79]. Every experimental group (unit), consisting of 10 individuals, of the same breed, age, and weight, was restricted within a pen, where feeding was applied at group level while ensuring adherence to welfare conditions. After consuming the plant-based feed, the pigs were allowed to go out into the paddock. A one-month interval was inserted between the plant experiments conducted on different animals. The diet was tailored to the animals based on their respective age categories (Table 4). The average daily feed intake per animal was as follows: 3 kg for sows, 2 kg for fatteners, and 0.7 kg for weaners, respectively. Each pig received a dosage of *A. sativum* of 180 mg/kg bw/day, which was divided into two portions and given for 10 consecutive days. *A. absinthium* was administered in a dosage of 90 mg/kg bw/day, divided into two portions, for the same period of time as garlic. Before initiating the treatment (day 0), a coproparasitological examination was conducted. Following therapy, two additional assessments (on days 14 and 28) were performed across all farms, plants, and age categories. The experiment began with a 28-day test of *A. sativum*, followed by an equal duration testing of *A. absinthium*. Both the feed and medicinal plants provided to the animals were certified by the producer.

**Table 4:** The diet of the experimental groups based on defined age groups

Feed	*A. sativum* group			*A. absinthium* group		
	**Weaners**	**Fatteners**	**Sows**	**Weaners**	**Fatteners**	**Sows**
Corn %	38.12	45.98	37.54	38.31	46.24	38.02
Barley %	30	12	20	30	12	20
Wheat %	20	25	25	20	25	25
Peas %	10	15	15	10	15	15
Calcium carbonate %	1.5	1.5	1.5	1.5	1.5	1.5
Garlic bulbs %	0.38	0.52	0.96	-	-	-
Aerial parts of wormwood %	-	-	-	0.19	0.26	0.48

Rectal faecal samples weighing approximately 15–20 g each, were collected individually, on days 0, 14 and 28 of the experiment, placed in clean containers, and were macroscopically examined for the presence of visible parasites. The samples were then stored at 2–8ºC for 24–48 h, until further examination. Stored samples were then examined using the following methods: centrifugal sedimentation, flotation—Willis method, faecal smear stained by modified Ziehl–Neelsen technique, Blagg method, McMaster egg counting technique, and in vitro nematode larvae/protozoan oocysts cultures (Additional file 3). The individual intensity was calculated using the McMaster quantitative faecal flotation technique. In contrast, the average intensity of parasitism was established as the arithmetic means of the counted eggs, cysts, or oocysts of a particular parasite species, divided by the number of individuals of the age group (*n* = 10) [8, 79–81].

### Assessment of antiparasitic efficacy

Faecal egg count reduction test (FECRT) was used to ascertain the antiparasitic efficacy of *A. sativum* and *A. absinthium* and faecal egg count reduction (FECR) was reported [82, 83]:** FECR (%) = 100 x (1–[T2/T1] x [C1/C2])**, where T1 and T2 are the mean pre- and post-treatment faecal egg counts (FEC) of a treated group, and C1 and C2 are the mean pre- and post-treatment FEC of control group. This formula was also utilized in a previous article [79].

### Statistical analysis

Data were analyzed with Statistica program (v. 13.5, TIBCO, Tusla, OK, USA). Excel® from Microsoft Office 365 was used to visually represent the raw data. Column graphs were used to represent the prevalence of parasites by farm, age group, and plant. The presence of *Cryptosporidium* spp. infection was reported as absolute frequency per age group, plant (A. *sativum*, A. *absinthium*), and farm. Due to the limited sample size for each farm, group (EG-experimental groups vs. CG-control group), and plant, as well as the high variability in the data, the count of parasites were reported as the median with the 25th percentile and 75th percentile range.

To compare the EG with the CG on each farm and investigated day (0, 14, and 28), it was employed the two-sided Mann–Whitney test at a significance level (α) of 5%. The effectiveness of the investigated plants was assessed using the two-sided Friedman test, followed by the Wilcoxon test whenever statistical significance was reached. The Bonferroni adjustment was applied to reduce the change of type 1 error α in post-hoc analysis, whenever Friedman test proved statistically significance. The significance level (α*) was adjusted based on the maximum possible number of parasites (intensity) per farm and age group, which was four in our study, resulting in α* = 1.25% (Additional file 4).

### Supplementary Information

**Additional file 1.** Ontologies/pathogens, diseases, medicinal plants and chemical compounds used in experiment. The ontologies utilized in this manuscript refers to a formal representation or conceptualization of parasitology and plants domains. They provide a structured method for representing the entities and the relationships that exists between them.

**Additional file 2.** Aspects regarding the HPLC–MS method used for the analysis of alcoholic plant extracts.

**Additional file 3.** Coproparasitological methods used for the diagnosis of parasitic infections.

**Additional file 4.** Methodological aspects regarding the statistical analysis of results.

## Data Availability

The raw data have been deposited in the repository of the University of Agricultural Sciences and Veterinary Medicine of Cluj-Napoca. The data that support the study are available from the corresponding author upon reasonable request.

## Abbreviations

*A. absinthium*: *Artemisia absinthium*
*A. sativum*: *Allium sativum*
*A. suum*: *Ascaris suum*
*B. coli*: *Balantioides coli*
*T. suis*: *Trichuris suis*
*S. ransomi*: *Strongyloides ransomi*
CG: Control group
EG: Experimental group
F1: Farm 1
F2: Farm 2
*T. muris*: *Trichuris muris*
*T. spiralis*: *Trichinella spiralis*
*H. gallinarum*: *Heterakis gallinarum*
FECRT: Faecal egg count reduction test
