# Supplementary material for: In vivo assessment of the antiparasitic effects of Allium sativum L. and Artemisia absinthium L. against gastrointestinal parasites in swine from low-input farms
Source: BMC Vet Res. 2024 Apr 1;20:126. doi: 10.1186/s12917-024-03983-3 (PMC10983701; doi:10.1186/s12917-024-03983-3)
Supplement: Supplementary file 1 — Additional file 1. Ontologies/pathogens, diseases, medicinal plants and chemical compounds used in experiment. The ontologies utilized in this manuscript refers to a formal representation or conceptualization of parasitology and plants domains. They provide a structured method for representing the entities and the relationships that exists between them. [file 12917_2024_3983_MOESM1_ESM.docx]

**Additional file 1** Ontologies/pathogens, diseases, medicinal plants and chemical compounds used in experiment.

| Traits | ATOL*, AHOL**, ChEBI*** , OPL****, IPNI*****, References |
| --- | --- |
| Parasite load traits | |
| Parasite Oocysts (OPG) | Oocyst Stage |
| Parasite Cysts | Cyst Stage |
| Parasite Eggs (EPG) | Egg Stage |
| Parasite used | |
| *Eimeria* spp. | AHOL_0004070 |
| *Balantidium coli* | AHOL_0004016 |
| *Cryptosporidium* spp. | AHOL_0004175 |
| *Ascaris suum* | AHOL_0004179 |
| *Trichuris suis* | AHOL_0004186 |
| *Strongyloides ransomi* | AHOL_0004178 |
| *Oesophagostomum* spp. | AHOL_0004181 |
| Disease description | |
| Ascaridiosis | AHOL_0005382 |
| Coccidiosis | AHOL_0005374 |
| Cryptosporidiosis | AHOL_0005377 |
| Medicinal plants used | |
| *Allium sativum* L. | 528796-1 |
| *Artemisia absinthium* L. | 300106-2 |
| Chemical compounds detected | |
| Polyphenols (μg/mL) | 26195 |
| Tocopherols (ng/mL | 135821 |
| Sterols (μg/mL) | 15889 |
| Methyoxylated flavones (ng/mL) | 25241 |
| Sulfoxide (μg/mL) | 22093 |
| Sesquiterpene lactones (ng/ml) | 37667 |

*Traits in reference to the ontology ATOL: [https://www.atol-o.com/en/atol-2/](https://www.atol-ontology.com/en/atol-2/); **Traits in reference to the ontology AHOL: [https://www.atol-onntologytology.com/ahol/](https://www.atol-ontology.com/ahol/); *** Chemical Entities of Biological Interest-ChEBI: <https://www.ebi.ac.uk/chebi/>; **** Ontology for Parasite Life cycle: <http://wiki.aiisc.ai/index.php/Ontology_for_Parasite_Life_Cycle>; ***** International Plant Names Index-IPNI : <https://www.ipni.org/p/3>.
