# Supplementary material for: In vivo assessment of the antiparasitic effects of Allium sativum L. and Artemisia absinthium L. against gastrointestinal parasites in swine from low-input farms
Source: BMC Vet Res. 2024 Apr 1;20:126. doi: 10.1186/s12917-024-03983-3 (PMC10983701; doi:10.1186/s12917-024-03983-3)
Supplement: Supplementary file 2 — Additional file 2. Aspects regarding the HPLC–MS method used for the analysis of alcoholic plant extracts. [file 12917_2024_3983_MOESM2_ESM.docx]

**Additional file 2** Aspects regarding the HPLC-MS method used for the analysis of alcoholic plant extracts.

HPLC-MS is a highly efficient analytical technique employed to ascertain the composition and purity of *A. sativum* and *A. absinthium* alcoholic extracts. This fusion of high-performance liquid chromatography (HPLC) and mass spectrometry (MS) presents powerful capabilities in both physical separation and mass analysis, thereby delivering precise insights into the composition of a given sample. The choice of ionization source and mode for MS system varied depending on the chemical class of each compound within the plant sample. Specifically, for the analysis of polyphenols and methoxylated flavones, the electrospray ionization source was utilized. In contrast, the atmospheric pressure chemical ionization source was used when analyzing tocopherols, sesquiterpene lactones, and sterols.
