# Supplementary material for: In vivo assessment of the antiparasitic effects of Allium sativum L. and Artemisia absinthium L. against gastrointestinal parasites in swine from low-input farms
Source: BMC Vet Res. 2024 Apr 1;20:126. doi: 10.1186/s12917-024-03983-3 (PMC10983701; doi:10.1186/s12917-024-03983-3)
Supplement: Supplementary file 3 — Additional file 3. Coproparasitological methods used for the diagnosis of parasitic infections. [file 12917_2024_3983_MOESM3_ESM.docx]

**Additional file 3** Coproparasitological methods used for the diagnosis of parasitic infections.

The Willis method is commonly used to concentrate parasite eggs (*A. suum*, *T. suis*, *S. ransomi*, *Oesophagostomum* spp.), oocysts (*Eimeria* spp.), and cysts (*B. coli*) from faecal samples. This technique entails homogenizing the faecal sample with a supersaturated sodium chloride solution, causing the parasitic elements to ascend to and float at the surface, thus being used for subsequent analysis. The McMaster method is a quantitative technique employed to assess the intensity of parasitic infections. Therefore, it aims to evaluate the therapeutic efficacy. The active sedimentation technique is employed for concentrating the eggs of *Fasciola hepatica*, *Dicrocoelium lanceolatum,* and *Macracanthorhynchus hirudinaceus*. Initially, the faecal samples are homogenized in low-density solutions, typically tap water, and then the concentration process is carried out through centrifugation. The modified Blagg technique is recommended for concentrating and highlighting *Giardia* spp. cysts. The modified Ziehl-Neelsen stained faecal smear is used to highlight the oocysts of *Cryptosporidium* spp., which are spherical in shape and appear as red on a green background.
