# Supplementary material for: In vivo assessment of the antiparasitic effects of Allium sativum L. and Artemisia absinthium L. against gastrointestinal parasites in swine from low-input farms
Source: BMC Vet Res. 2024 Apr 1;20:126. doi: 10.1186/s12917-024-03983-3 (PMC10983701; doi:10.1186/s12917-024-03983-3)
Supplement: Supplementary file 4 — Additional file 4. Methodological aspects regarding the statistical analysis of results. [file 12917_2024_3983_MOESM4_ESM.docx]

**Additional file 4** Methodological aspects regarding the statistical analysis of results.

The Mann–Whitney test was selected due to the non-parametric nature of our data. This test is particularly effective when the data doesn’t meet the assumptions of normality, which was evident from preliminary checks on our dataset. Our data distribution was verified using the Shapiro-Wilk test, which indicated a non-normal distribution, thereby justifying the use of the Mann-Whitney test. The Friedman test, a non-parametric alternative to the one-way ANOVA with repeated measures, was applied to test the effectiveness of the investigated plants over different periods. Since our data involved repeated measurements (on days 0, 14, and 28) and didn't meet the sphericity assumption (as tested by Mauchly's Test of Sphericity), the Friedman test was deemed appropriate.

Assumptions and Validations: Before applying the Mann-Whitney and Friedman tests, we ensured that our data met the required assumptions for each test like independence of observations and the use of ordinal variables. When post-hoc analyses were necessary following the Friedman test, the Wilcoxon test was applied. We used the Bonferroni adjustment to correct for the increased risk of type 1 error due to multiple comparisons.
